# Dental Meeting

**Published:** 1891-11

**Authors:** 


### Dental Meeting.

The twenty-third annual union meeting of the Fifth, Sixth, Seventh and Eighth District Societies of New York State, was held in Buffalo on the 27th, 28th and 29th of October. These union meetings, being held annually, enable the different district societies to keep in touch, and to maintain an acquaintance and friendship that is not only pleasant but profitable. Quite a number of papers were read and freely discussed. The first one "Treatment and Destruction of the Pulp," by Dr. Frank French, of Rochester. The subject was discussed by quite a large number of those present. The second paper, on "Antral Abscesses," was read by Dr. M. O. Cooley, of Niagara Falls. The third paper, on "Cause and Effect," was read by S. B. Palmer, of Syracuse; this also elicited an interesting discussion. Quite an exhaustive paper on "The Mouth and Connecting Cavities in Relation to Speech" was read by Dr. A. L. Benedick, of Buffalo. This paper was illustrated with charts—a skull and various anatomical preparations. A consideration of this paper proved very interesting.

The forenoon of the second day was wholly occupied with a series of very interesting clinics and demonstrations including Crown and Bridge Work, Special Methods of Filling and Treatment of Exposed Pulps. Quite a number of new and interesting points were bronght out in the morning's clinic. The afternoon session was devoted to reading papers, the first of which was one by Dr. Barrett on "Examining Boards vs. Dental Colleges." The paper elicited considerable discussion in which quite a number of members participated. Following this was a paper by Dr. W. H. Bergtold, of Buffalo, on "The Mouth as a Source of Infection." Many interesting facts were presented in the paper. After this a third paper on "Immediate Root Filling" was read by Dr. R. H. Hofheinz, of Rochester; those who spoke on the subject concurred in the main with the positions taken by the paper.

The annual dinner of the convention took place at the Hotel Iroquois, at 8 o'clock in the evening, with all the usual accompaniments of a very fine spread, with quite an elaborate list of toasts, responses and a good social time generally, the banquet closing at the good and seasonable hour of eleven o'clock.

The next morning at nine o'clock, a session was held, at which Dr. I. C. Curtis, read a paper on "The Relative Value of Cohesive and Non-Cohesive Gold, and a Combination of the Two;" this paper elicited quite an extended and general discussion. Dr. L. G. Curtis, of Syracuse, read a paper entitled "Where Dentistry looks over into Oral Surgery." This paper was also freely discussed. Dr. W. F. Lowe presided over the meetings with marked dignity and success. For the very thorough manner in which the arrangements were made and executed, the body was in a large measure indebted to Dr. C. S. Butler, of Buffalo, and his associate committee-men.

An invitation was extended by the Sixth District Society to the other societies, to hold the next annual meeting in Binghampton; and this no doubt will be the place of holding the next meeting. The union meetings of these District Societies serve as a bond of affiliation, and as a means of acquaintance and good fellowship, the value of which it is hard to estimate. There were quite a number of dentists from other States present, all of whom expressed themselves as delighted with what they saw and heard, and the only regret that was heard was, that more visitors were not present to enjoy the pleasures of the occasion.

